# Transient Thermal Conductivity Minimum in Phenolic Foam Insulation: Closed-Cell Structural Dependence and Long-Term Aging Behavior

**DOI:** 10.3390/polym18151862

**Published:** 2026-07-29

**Authors:** Minjung Bae, Jaesik Kang, Hosang Ahn

**Affiliations:** Department of Building Energy Research, Korea Institute of Civil Engineering and Building Technology, Goyang 10223, Republic of Korea; jskang@kict.re.kr (J.K.); hahn@kict.re.kr (H.A.)

**Keywords:** phenolic foam, thermal conductivity aging, transient thermal conductivity minimum, closed-cell foam insulation, n-pentane redistribution

## Abstract

Closed-cell phenolic foam (PF) insulation is widely assumed to exhibit monotonically increasing thermal conductivity after cutting, driven by progressive blowing agent release. This study reports a previously uncharacterized behavior: a consistent decrease–minimum–rebound pattern in the days to weeks after cutting, defined here as the transient thermal conductivity minimum (TTCM). Using n-pentane-blown PF foam, thermal conductivity and mass were tracked under standard (23 °C) and accelerated aging conditions (70 °C, 110 °C; slicing per KS M ISO 11561), complemented by gas chromatography–flame ionization detection (GC-FID), attenuated total reflectance Fourier-transform infrared spectroscopy (ATR-FT-IR), thermogravimetric analysis/differential scanning calorimetry (TGA/DSC), and scanning electron microscopy (SEM). TTCM reductions of 3.8–16.7% were observed across all closed-cell specimens; a reference PF foam with cell-wall micro-perforations showed no TTCM, establishing intact closed-cell structure as a prerequisite. The mechanism is attributed to redistribution of dissolved n-pentane from the polymer matrix into the cell gas phase, thermodynamically favored by the proximity of n-pentane’s boiling point (36.07 °C) to the heat flow meter (HFM) hot plate temperature (33 °C). In the post-TTCM phase, thermal conductivity rises progressively as n-pentane is displaced by air; the rate of this rise is strongly dependent on diffusion path length. These results establish two mechanistically distinct aging stages and identify their respective governing factors.

## 1. Introduction

Closed-cell polymeric foam insulation materials including polyurethane (PUR), polyisocyanurate (PIR), and phenolic foam (PF) achieve their thermal performance by retaining blowing agent gas within sealed cells and typically exhibit thermal conductivity values in the range of 0.018–0.025 W/(m·K) [1,2]. This performance, however, is not fixed at the time of manufacture. Blowing agent molecules gradually permeate through polymer cell walls and escape to the surroundings, while air components diffuse inward to replace them [3,4]. The resulting shift in cell gas composition causes the thermal conductivity of the foam to increase progressively over time.

This phenomenon has been studied primarily in PUR and PIR foams [1,4]. The concurrent outward diffusion of blowing agent and inward diffusion of air follows Fick’s law [5] and is accompanied by a gradual reduction in material mass as cell gas composition evolves [6,7]. The rate of this diffusion depends on the type of blowing agent, specimen thickness, foam density, and the nature of the facing material [3,5]. Focusing specifically on the role of facings, Mukhopadhyaya et al. [8,9] tracked PIR foam specimens with different facing types over extended periods and experimentally confirmed that the gas barrier performance of the facing is critical to maintaining thermal resistance. Andersons et al. [10] quantified this rate dependence by back-calculating effective diffusion coefficients from thermal aging time-series data. Berardi and Madzarevic [11] used gas chromatography–flame ionization detection (GC-FID) analysis to demonstrate that the gradual change in cell gas composition directly drives the rise in thermal conductivity.

PF foam is a thermoset polymeric foam produced by acid-catalyzed curing of resole-type phenol-formaldehyde resin. Pentane-series hydrocarbons, such as n-pentane, cyclopentane, and isopentane, are used as blowing agents, and these compounds exist simultaneously in two states: dissolved and adsorbed within the polymer matrix, and as vapor in the cell gas phase [12]. Hong et al. [12] reported that the partitioning of blowing agent between these two phases in polyurethane foam is temperature-dependent, and a similar mechanism is likely operative in PF foams where n-pentane serves as the blowing agent. Because the thermal conductivity of blowing agent gas (0.013–0.015 W/(m·K)) is substantially lower than that of air (0.026 W/(m·K)), the extent to which the blowing agent is retained within the cells governs insulation performance [13,14]. Water is produced as a by-product of the curing reaction and remains in the foam matrix [14,15]; this residual moisture (thermal conductivity approximately 0.016 W/(m·K)) may contribute secondarily to the effective thermal conductivity of the cell gas mixture [16]. Separately, Pilon et al. [17] showed theoretically that the timescale of gas diffusion in closed-cell foams scales with the square of the diffusion path length. Together, these studies provide the theoretical framework needed to interpret the initial and long-term thermal conductivity behavior of PF foams blown with n-pentane.

The methylene bridges of resole resins are known to undergo oxidative degradation pathways involving carbonyl group formation. Chen et al. [18] used thermogravimetric analysis coupled with Fourier-transform infrared spectroscopy (FT-IR) to demonstrate that methylene bridge cleavage is a primary thermal degradation pathway in PF resins. Costa et al. [19] subjected PF resins with varying degrees of crosslinking to thermal treatment in air and systematically characterized the relationship between crosslink structure and oxidative degradation behavior. Pintus et al. [20] confirmed that methylene bridge oxidation in naturally aged PF foam occurs alongside morphological changes in cell structure. Yu et al. [21] showed by Attenuated total reflectance Fourier-transform infrared spectroscopy (ATR-FT-IR) analysis that the relative contributions of oxidation, crosslinking, and hydrolysis vary with aging conditions, while Leem et al. [22] experimentally demonstrated that long-term water vapor exposure alters the PF resin structure and affects physicochemical properties including thermal conductivity. These studies collectively suggest that oxidative degradation of the PF resin matrix may contribute to changes in long-term thermal properties.

PF foam differs fundamentally from PUR and PIR systems in resin chemistry, blowing agent characteristics, and the presence of residual moisture from the curing reaction. How these material-specific differences manifest in thermal conductivity behavior following cutting has not been systematically examined. A prior study compared thermally and slicing-accelerated aging protocols across extruded polystyrenes (XPS), PIR, and PF foams [23], but variation in thermal conductivity behavior by through-thickness sampling position was not addressed. The present study focuses on both aspects.

The first aspect concerns the governing factors of the initial post-cutting thermal conductivity change and its dependence on conditions. In PUR and PIR foams, air ingress and blowing agent egress proceed simultaneously from the moment of cutting, causing rapid shifts in cell gas composition and concurrent mass loss [6,7]. In PF foam, the distinctive boiling point characteristics of pentane-series blowing agents and the presence of residual moisture create conditions in which a different initial behavior may emerge; yet no study has systematically documented this [14]. Characterizing this initial behavior is relevant to the interpretation of accelerated aging test results [24,25]. To this end, aging conditions in the present study were selected based on established domestic and international standards for evaluating the long-term thermal performance of foam insulation: thermal acceleration at 70 °C and 110 °C in accordance with BS EN 13166 Annex C [2], and slicing acceleration in accordance with KS M ISO 11561 Method B [25]. These standardized protocols were adopted to ensure that the experimental conditions reflect those used in practice for long-term thermal performance assessment of PF insulation, and to enable direct comparison with results obtained under equivalent conditions in prior studies.

The second aspect concerns differences in thermal conductivity evolution according to through-thickness sampling position. Structural and chemical gradients develop between the surface layers and the core during foam manufacture [26], but how these gradients are reflected in long-term thermal conductivity behavior has not been systematically examined. The present study explores these differences through thermal conductivity time-series data and chemical aging indicators.

Against this background, the present study pursues three objectives. First, thermal conductivity time-series data were collected after cutting PF foam insulation specimens under varying temperature conditions, cutting timing, and specimen thickness; these were interpreted in conjunction with gravimetric data and residual blowing agent concentrations to identify the dominant factors governing thermal conductivity change across the initial and long-term phases. A PF reference foam exhibiting dispersed cell-wall micro-perforations was included to examine the influence of cell structure on these dynamics. Second, GC-FID blowing agent analysis and ATR-FT-IR oxidation indicators were integrated with thermal conductivity and mass change data to explore the physical and chemical mechanisms underlying thermal conductivity change in each phase. Third, differences in blowing agent distribution, resin chemical structure, and thermal conductivity evolution as a function of through-thickness sampling position were examined, and the study assessed whether these variables exhibit a consistent directional pattern along the common axis of through-thickness position.

## 2. Materials and Methods

### 2.1. Materials

#### 2.1.1. Closed-Cell PF Insulation Specimens

PF foam insulation boards were sourced from a single domestic manufacturer. The boards are produced using resole-type phenol-formaldehyde resin as the polymer matrix, with n-pentane as the blowing agent. The apparent density of the intact board, including facings, is approximately 38.8 kg/m^3^; with facings removed, the foam core density ranges from approximately 35.6 to 36.7 kg/m^3^. Closed-cell content, measured in triplicate according to ASTM D6226-15 [27], was 98.3%, 96.7%, and 99.2%, yielding a mean of 98.1%. Specimens were prepared in two groups: one for long-term and accelerated aging studies, and one for short-term thermal conductivity tracking and residual blowing agent concentration analysis. Lots A, B, and C, produced between 2020 and 2021, were used for the aging studies, while a single production lot manufactured on 29 May 2024 (Lot 240529) was used for the short-term tracking and blowing agent analysis.

Specimens for long-term thermal performance evaluation were prepared from Lots A, B, and C, produced between August 2020 and April 2021. Immediately after production, facings (aluminum foil and nonwoven fabric) were removed, and specimens were stored in a temperature- and humidity-controlled room without sealing, exposed to ambient air throughout natural aging. Storage conditions were maintained within 22.7–24.9 °C and 49–58% relative humidity. Standard non-accelerated aging and three types of accelerated aging were applied; the specimen configuration for each condition is summarized in Table 1. Lot-C-Std was tested with its aluminum foil and nonwoven facings intact, to track the long-term gas barrier effect of the facing. Among the long-term aging specimens, the core-region sample (A-Core) and surface-region samples (B-Surf, C-Surf) were prepared exclusively for ATR-FT-IR analysis to examine the through-thickness distribution of chemical aging; sampling procedures are described in Section 2.4.2. Scanning electron microscopy (SEM) observation was performed on Lot-C specimens to characterize closed-cell morphology. Thermogravimetric analysis (TGA) and differential scanning calorimetry (DSC) thermal stability analyses were conducted on Lot-C-Std; details are provided in Section 2.2.

Short-term tracking specimens (Lot 240529) were prepared from thirteen full boards (80 × 1200 × 2000 mm) sealed in a pallet unit at the time of preparation. The boards have an asymmetric facing configuration, with aluminum foil on the upper face and nonwoven fabric on the lower face. Specimens were classified into three types, namely Core, Surf, and Board, based on their through-thickness sampling position and facing treatment. Core specimens were prepared by removing material from the upper and lower surface layers to retain only the central region, representing the core of the board. Surf specimens were taken from approximately the top 50 mm beneath the aluminum foil facing, representing the surface region. These two specimen types differ in their through-thickness sampling position; in both cases, facings were completely removed prior to measurement. Board specimens were measured in their intact board state with facings retained, providing a thermal conductivity reference value close to the as-manufactured condition. Thermal conductivity measurement of Board specimens commenced on Day 544 post-production, the day the board was cut, which was defined as Day 0 for the Board series. Board specimens are used in Section 3.1.1 to quantify the initial post-cutting thermal conductivity behavior, and given their distinct storage history under long-term sealed conditions, are not used for direct comparison with Core and Surf specimens.

CP-series specimens, cut from the same lot (Lot 240529) after different sealed storage durations, were prepared exclusively for GC-FID analysis of residual blowing agent concentrations as a function of the time elapsed between production and cutting. CP-0 was cut immediately after production, while CP-34, CP-105, and CP-544 were kept as sealed, intact boards for the respective number of days before cutting. After cutting, all specimens were double sealed in polyethylene bags and aluminum foil to minimize exposure to ambient air prior to analysis; differences in chemical state among specimens are attributable to differences in sealed storage duration. Facings were retained throughout storage and removed immediately before GC-FID analysis. CP-544 shares the same lot and sealed storage history as the Board specimens and was used as the reference value for residual blowing agent concentration at the time of cutting. Detailed characteristics are summarized in Table 2.

#### 2.1.2. Reference PF Specimens

To isolate the influence of cell morphology on thermal conductivity behavior, a reference PF foam exhibiting cell-wall micro-perforations (Ref-OC), sourced from a different manufacturer, was included. This material was produced on 28 April 2021, and two specimens were prepared: a standard aging specimen (Ref-OC-Std) and a slicing-accelerated specimen (Ref-OC-Slicing). Details are summarized in Table 3. Both specimens were tracked for thermal conductivity and mass using the same procedures applied to the closed-cell specimens. Closed-cell content measured according to ASTM D6226-15 [27] was 98.1% for the closed-cell specimen (Lot-C-Std) and 95.0% for Ref-OC, confirming a quantitative difference in cell structure between the two materials. As a product from a different manufacturer, this specimen may differ in resin formulation and processing conditions. However, differences in cell structure were confirmed through SEM observation and ASTM D6226-15 closed-cell content measurement, which formed the basis for its use as a reference material.

**Figure 1:**
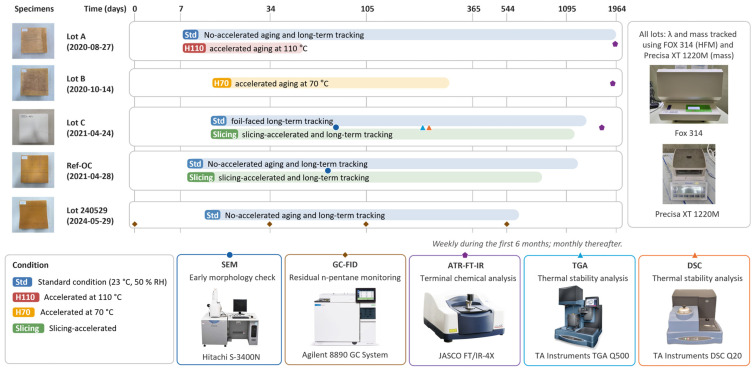
Overview of experimental design showing specimen groups, aging conditions (standard 23 °C, 70 °C accelerated, 110 °C accelerated, and slicing), measurement timeline, and analytical instruments employed in this study.

A comprehensive overview of all specimen groups, aging conditions, measurement periods, and analytical instruments is provided in Figure 1.

### 2.2. Accelerated and Standard Aging Conditions

Three accelerated aging protocols were applied to compress the long-term degradation behavior of the foam into an experimentally accessible timeframe. These methods were selected based on domestic and international standards for evaluating the long-term thermal performance of foam insulation.

Slicing-accelerated aging was conducted in accordance with KS M ISO 11561 Method B [25]. Specimens were cut to a thickness of 10 ± 1 mm immediately upon receipt and reassembled in their original stacking order within three hours. The first thermal conductivity measurement was taken within eight hours of cutting, and the reassembled stack was stored at 23 ± 2 °C and 50 ± 5% relative humidity. Although the standard specifies only a single measurement at 91 ± 7 days, the present study performed measurements at approximately weekly intervals up to 90 days post-slicing. An additional measurement was also taken at approximately Day 1739 to document long-term behavior.

Thermal accelerated aging at 70 °C was performed in accordance with BS EN 13166 Annex C Method 2 [2]. Specimens were aged at 70 ± 2 °C and thermal conductivity was measured at approximately seven-day intervals. At each measurement point, specimens were removed from the aging environment and conditioned at 23 ± 2 °C and 50 ± 5% relative humidity until mass equilibrium was reached, defined as a mass change of less than 0.1% within 24 h. Although the standard specifies an aging duration of 175 ± 5 days, aging in the present study was extended to approximately 235 days.

Thermally accelerated aging at 110 °C was performed in accordance with BS EN 13166 Annex C Method 1 [2]. Specimens were aged at 110 ± 2 °C; measurement intervals, conditioning criteria, and transfer procedures were identical to those applied in the 70 °C protocol.

To verify the thermal stability of the PF resin skeleton under 110 °C accelerated aging conditions, TGA and DSC analyses were performed on Lot-C-Std specimens by KOPTRI under commission. As the accelerated aging specimens (Lot-A-H110, Lot-B-H70) originate from the same manufacturer and product as Lot-C-Std, their resin compositions are considered equivalent, and these analytical results are applied accordingly. TGA was performed using a TGA Q500 (TA Instruments, New Castle, DE, USA) at a heating rate of 20 °C/min under an air atmosphere, from ambient temperature to 800 °C. DSC was performed using a DSC Q20 (TA Instruments, New Castle, DE, USA) at a heating rate of 20 °C/min, from −10 °C to 230 °C. Results and their interpretation are presented in Section 3.1.2.

Standard aging was conducted in a temperature- and humidity-controlled room. Specimens were stored without sealing, and environmental conditions were recorded on a monthly basis and maintained within 22.7–24.9 °C and 49–58% relative humidity.

### 2.3. Thermal Conductivity and Mass Tracking

Thermal conductivity was measured using a heat flow meter (HFM) apparatus, the FOX 314 (TA Instruments, New Castle, DE, USA), in accordance with KS L 9016 [28]. Consistent measurement conditions were applied throughout: hot plate at 33 °C, cold plate at 13 °C, mean temperature 23 °C, and temperature difference 20 °C. Measurement repeatability for the same specimen was within ±1%. A single specimen was tracked per condition over the long term; independent replicate specimens for statistical assessment were not available. Accordingly, specimen-to-specimen variability cannot be statistically quantified, and the interpretation of results is limited to trend analysis based on single-specimen tracking.

For short-term tracking specimens (Lot 240529), the first measurement of Core and Surf specimens was taken on the day of cutting (Day 0). For Board specimens, measurement commenced on Day 544 post-production, the day of cutting, which was defined as Day 0 for the Board series. Measurements were taken at intervals of several days up to approximately Day 60, and approximately monthly thereafter. For long-term aging specimens (Lots A, B, and C), the first measurement was taken 7–8 days after cutting. Measurements were taken approximately weekly during the first six months and approximately monthly thereafter; standard-conditioning specimens were tracked for up to approximately 1900 days.

Specimen mass was recorded simultaneously with thermal conductivity measurements using a precision balance (Precisa XT 1220M, Precisa Gravimetrics AG, Dietikon, Switzerland). Before each measurement, accelerated aging specimens were removed from the aging environment and conditioned at 23 ± 2 °C and 50 ± 5% relative humidity until mass equilibrium was reached.

### 2.4. Chemical and Structural Analysis

#### 2.4.1. GC-FID Blowing Agent Analysis

Residual n-pentane in the foam matrix was analyzed by GC-FID, commissioned to KOPTRI. Four specimens constituting the cutting time series (CP-0, CP-34, CP-105, and CP-544) were analyzed. Each specimen was sampled from the planar center of an 80 × 1200 × 2000 mm board over a 160 × 80 mm area, and then sectioned in the thickness direction into the following layers: an upper zone adjacent to the aluminum foil facing (AL, 20 mm), a discarded intermediate zone (10 mm), a central zone (CORE, 20 mm), another discarded intermediate zone (10 mm), and a lower zone adjacent to the nonwoven facing (NO-AL, 20 mm). The intermediate zones were excluded from analysis to prevent cross-contamination between adjacent layers. Immediately after cutting, each sectioned specimen was wrapped in aluminum foil and sealed in a polyethylene bag to prevent volatile loss. Analysis was performed using an Agilent 8890 gas chromatograph (Agilent Technologies Inc., Santa Clara, CA, USA), with quantification based on an external calibration curve prepared from analytical-grade n-pentane standards. The calibration curve was constructed from five standards in the concentration range of 1.0–100.0 mg/L, with a coefficient of determination (R^2^) of 0.9997, indicating excellent linearity. Cyclopentane was analyzed under the same conditions but was not detected in any specimen.

#### 2.4.2. ATR-FT-IR Resin Structural Analysis

ATR-FT-IR analysis was performed to assess chemical changes in the resole resin matrix resulting from long-term natural aging. Samples A-Core, B-Surf, and C-Surf were collected from the core and surface regions of long-term naturally aged specimens, and all measurements were conducted at a single time point on 22 December 2025. At that time, the aging duration of each batch ranged from 1697 to 1932 days. Samples measuring 10 × 10 mm were taken from each region: A-Core from the core region, and B-Surf and C-Surf from the surface region. Analysis was performed using an FT-IR spectrometer equipped with a germanium (Ge) ATR crystal (JASCO FT/IR-4X, JASCO Corporation, Tokyo, Japan). Spectra were acquired over the range of 700–4000 cm^−1^ at a resolution of 4 cm^−1^ with 32 accumulations.

Transmittance spectra were converted to absorbance (A = −log T/100) before analysis. Absolute peak intensities are subject to inter-specimen variability arising from differences in contact conditions between the ATR crystal and the sample surface. Therefore, peak intensities were calculated as baseline-corrected peak heights and normalized against the aromatic ring C=C stretching vibration at 1600 cm^−1^ as an internal reference, yielding relative intensities (I_x_). This normalization approach follows established practice in ATR-FT-IR studies of phenol-formaldehyde resins [29,30]. The spectral indices used in this study are: the para-substituted benzene ring C-H out-of-plane bending vibration at 815 cm^−1^ (I_815_) as an indicator of cured PF resin structure [19,29]; the carbonyl group stretching vibration at 1650 cm^−1^ (I_1650_) as an oxidation indicator [18]; and the CH_2_ scissoring vibration of methylene bridges at 1475 cm^−1^ (I_1475_) [29,30]. ATR-FT-IR is a semi-quantitative technique; in the present study, it was used for trend analysis through relative comparison of peak intensities across specimens. Each sample was measured once at a single location; precision was not verified through repeated measurements at multiple positions within the same sample. It should be noted that B-Surf was taken from the surface region of Lot-B, whereas A-Core originates from the core region of Lot-A and C-Surf from the surface region of Lot-C. Because the samples differ in both production batch and through-thickness position, batch effects and positional effects cannot be independently separated. This constitutes a methodological limitation of the present study; results are therefore interpreted only at the level of directional consistency, without causal attribution to either batch or position.

#### 2.4.3. Cell Morphology Observation

Scanning electron microscopy (SEM) observation was performed to compare the cell morphology of the closed-cell PF specimen (Lot-C) and the reference material (Ref-OC). Specimens were fractured to expose internal cell structure, mounted on aluminum stubs, and sputter-coated with platinum to a nominal thickness of approximately 10 nm. Observation was carried out using a scanning electron microscope (S-3400N, Hitachi High-Tech Corporation, Tokyo, Japan) operated at an accelerating voltage of 15 kV. The closed-cell specimen (Lot-C) was observed at ×270 magnification on Day 82 post-production, and the reference specimen (Ref-OC-Std) at ×210 magnification on Day 78 post-production. Both specimens were analyzed on 15 July 2021, using the same instrument.

## 3. Results and Discussion

### 3.1. Transient Thermal Conductivity Minimum (TTCM)

Prior studies have reported that the thermal conductivity of closed-cell PF foam rises continuously over time following cutting, driven by the progressive release of blowing agent and its replacement by air [3,9]. Periodic tracking from the moment of cutting, however, revealed a distinct initial behavior. Across all specimens, thermal conductivity declined for a period of several days to several weeks after cutting, passed through a transient minimum, and then transitioned into a sustained rise. This study designates the initial decline and minimum as the Transient Thermal Conductivity Minimum (TTCM).

Section 3.1.1 quantifies the TTCM and establishes through four independent lines of evidence that it cannot be attributed to instrumental artifacts of the HFM. Section 3.1.2 examines the physical processes responsible for the TTCM under standard and elevated-temperature conditions.

#### 3.1.1. TTCM Quantification and Instrumental Artifact Exclusion

**Figure 2:**
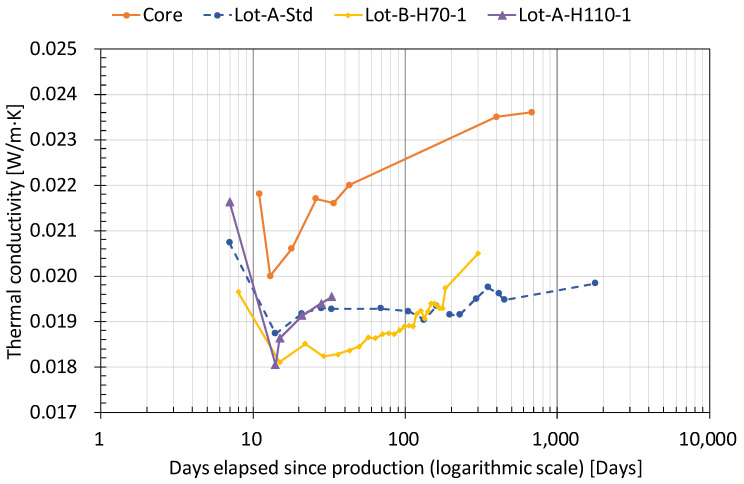
Early-stage thermal conductivity evolution of representative PF specimens under standard (23 °C), 70 °C accelerated, 110 °C accelerated, and short-term tracking conditions, plotted on a logarithmic time scale.

Figure 2 presents the early-stage thermal conductivity series for four representative specimens aged under different conditions: standard aging (Lot-A-Std), 70 °C accelerated aging (Lot-B-H70-1), 110 °C accelerated aging (Lot-A-H110-1), and short-term tracking (Core). In all four cases, thermal conductivity decreased over an initial period after cutting, passed through a minimum, and subsequently increased.

Lot-A-Std declined from 0.0207 W/(m·K) on Day 7 to 0.0187 W/(m·K) on Day 14, a reduction of 9.7%, before transitioning to a rise. Core decreased from 0.0218 W/(m·K) on Day 11 to 0.0200 W/(m·K) on Day 13 before turning upward. The same pattern held under elevated-temperature conditions: Lot-B-H70-1 decreased by 8.1% from 0.0197 W/(m·K) on Day 8 to 0.0181 W/(m·K) on Day 15, while Lot-A-H110-1 decreased by 16.7% from 0.0216 W/(m·K) on Day 7 to 0.0180 W/(m·K) on Day 14.

The consistent appearance of this pattern across four specimens subjected to different aging conditions and measured from different starting points suggests that the TTCM is not an isolated or incidental measurement artifact.

Table 4 summarizes the TTCM reduction magnitude and day of minimum across all standard and accelerated aging specimens. All measurements were conducted using the same HFM (FOX 314) under identical operating conditions: hot plate 33 °C, cold plate 13 °C, mean temperature 23 °C. With instrument and temperature conditions held constant, the only independent variable is the internal state of each specimen.

The Board specimen was cut 544 days after production, so the day elapsed since production and the day elapsed since cutting do not coincide. Table 4 presents both reference points accordingly. All other specimens were cut immediately after production or after only short-term storage, so the two references are effectively equivalent.

All values in Table 4 are single-specimen measurements; as noted in Section 2.3, independent replicates were not available. The reduction magnitudes are therefore reported as observed values without statistical verification, and their interpretation relies on the directional consistency seen across the full set of specimens and conditions.

Under identical instrument and measurement conditions, the TTCM reduction magnitude varied from 3.8% to 16.7%, a more than fourfold range across specimens (Table 4). If the reduction were attributable to instrument characteristics, similar magnitudes would be expected regardless of the specimen; instead, clear differences are observed between specimens. This variation points to the reduction magnitude depending on the internal state of each specimen rather than on measurement error.

The Board specimen was measured with its aluminum foil and nonwoven fabric facings intact, yet the TTCM still appeared, and the day on which the minimum was reached (Day 14 after cutting) was similar to that of specimens from which facings had been removed. Aluminum foil is known to have extremely low permeability to low-boiling-point organic gases such as n-pentane, and functions as a gas barrier in closed-cell foam insulation [8,9]. If mass transport to the exterior were the cause of the TTCM, the aluminum foil facing would be expected to impede gas movement, delaying the onset of the minimum or reducing the magnitude of the decline. No such difference was observed in the Board specimen, which suggests that the TTCM is driven by processes occurring within the specimen rather than by outward mass transport across the facing.

In Lot-C-Slicing, the TTCM lasted approximately one day, markedly shorter than the 13–25 days observed in full-board specimens of 50–80 mm thickness. This specimen was prepared by slicing the original board to approximately 10 mm thickness in accordance with KS M ISO 11561 Method B and restacking the slices. The slicing process opened cells adjacent to the cut surfaces and exposed cells that had been fully closed within the board interior to gas exchange with the surrounding atmosphere. The number of intact closed cells was thus reduced, and the diffusion path from the interior of each remaining closed cell to the outside was substantially shortened compared with the original board. The extreme compression of the TTCM duration indicates that the degree of closed-cell structural integrity influences the TTCM behavior.

**Figure 3:**
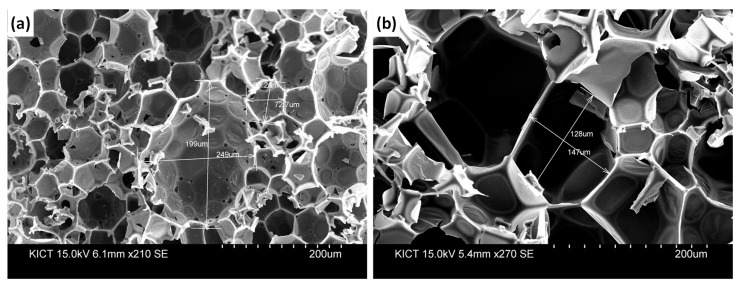
SEM micrographs comparing cell morphology of open-cell PF reference (Ref-OC) and closed-cell PF (Lot-C). (**a**) Ref-OC (×210) showing perforated cell walls with openings of varying sizes, enabling gas exchange between adjacent cells. (**b**) Lot-C (×270) showing intact, unperforated cell wall membranes that isolate each cell and retain the enclosed gas.

If the TTCM is associated with closed-cell structural integrity, it would not be expected to appear in a specimen that is structurally incapable of retaining gas. To examine this, a reference PF foam from a different manufacturer exhibiting dispersed cell-wall micro-perforations (Ref-OC) was introduced. Figure 3 compares the cell morphology of Ref-OC and Lot-C using SEM. In Ref-OC, fenestrations of varying sizes are distributed across the cell walls, providing direct gas transport pathways between adjacent cells. Lot-C, by contrast, exhibits continuous and intact cell wall membranes that isolate each cell. In closed-cell foams, gas diffusion is governed by permeation through the polymer cell wall membrane, and the effective diffusion coefficient is proportional to the membrane permeability [3,17]. As long as cell walls remain intact, this membrane resistance is the primary bottleneck for gas transport. When fenestrations are present, however, the permeation step is bypassed entirely, and gas moves directly between adjacent cells. As a result, gas transport is substantially enhanced in fenestrated structures, and the cell gas rapidly equilibrates with the surroundings.

These morphological differences are also supported quantitatively. The mean cell diameter was 139.6 ± 34.2 μm (*n* = 13) for Lot-C and 73.7 ± 16.1 μm (*n* = 13) for Ref-OC, indicating that the two specimens differ in cell size as well. The fenestrations observed in the Ref-OC cell walls had a mean diameter of 6.28 ± 1.93 μm (*n* = 14), a feature not observed in Lot-C. This difference in cell-wall structure is further corroborated by the closed-cell content measured in accordance with ASTM D6226-15, which was 98.1% for Lot-C and 95.0% for Ref-OC. Collectively, these results demonstrate that the presence of micro-perforations in the cell wall translates into a quantitative difference in the actual gas-sealing capacity of the two specimens.

**Figure 4:**
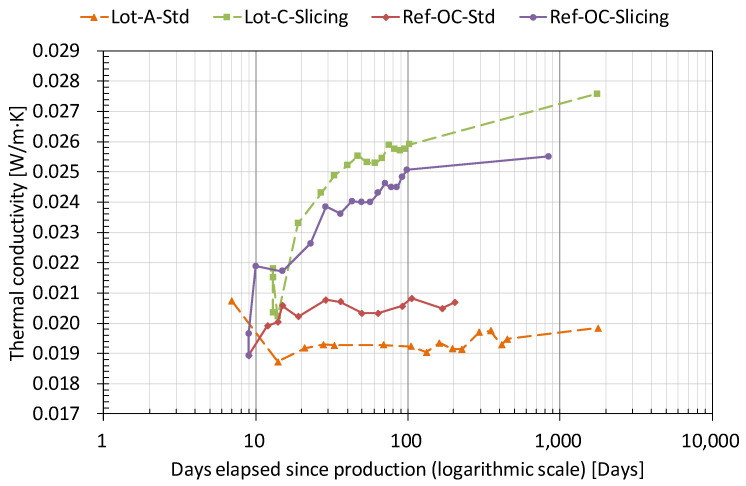
Thermal conductivity (λ) evolution of four specimens representing a structural spectrum of closed-cell integrity, plotted on a logarithmic time scale.

Figure 4 presents the thermal conductivity time series of Ref-OC and four Lot-C specimens on a logarithmic time scale. Under the same instrument and measurement conditions, the TTCM was absent from Ref-OC under all configurations. Ref-OC-Std exhibited a continuous, monotonic rise from Day 9 to Day 1720 with no declining phase, and Ref-OC-Slicing increased immediately from the day of slicing.

Because Ref-OC is a product from a different manufacturer, the possibility that the absence of the TTCM reflects differences in blowing agent type or concentration rather than cell structure differences must be considered. The thermal conductivity of closed-cell foam is highly sensitive to cell gas composition; if the blowing agent concentration were negligibly low, no rapid change in thermal conductivity would be expected even after slicing. However, thermal conductivity in Ref-OC-Slicing rose by 11.3% to 0.0219 W/(m·K) the day after slicing, and a sustained long-term rise was observed in Ref-OC-Std as well. These results indicate that sufficient releasable gas is present within the Ref-OC cells. The absence of the TTCM is therefore not attributable to differences in blowing agent concentration, but rather to the reduced capacity for gas retention associated with the cell-wall micro-perforations directly observed in Figure 3.

Taken together, these four observations point consistently in the same direction: the TTCM is a phenomenon arising from processes within the specimen, not a characteristic of the HFM instrument. The processes responsible are examined in Section 3.1.2.

#### 3.1.2. Closed-Cell Structural Integrity and Governing Factor Analysis

Section 3.1.1 established that the TTCM depends on closed-cell structural integrity and is driven by processes occurring within the cells. This section examines what specifically changes inside the cells, beginning with specimens under standard conditions and then turning to the effects of elevated temperature.

Under standard conditions, we examine in turn the interphase redistribution of n-pentane as the primary factor, followed by the possible contribution of residual moisture as a secondary factor. Under standard conditions (Lot-A-Std), mass change during the TTCM phase was only −0.2%, while thermal conductivity declined by 9.7% over the same period, a discrepancy of approximately 50-fold. This mismatch indicates that the process responsible for the TTCM under standard conditions involves changes within the cells rather than outward mass transport.

The candidate governing factors are examined in turn. The leading candidate is intracellular redistribution of n-pentane between phases. In closed-cell foams, blowing agent coexists in two phases simultaneously, dissolved within the polymer matrix and as vapor in the cell gas phase, and the partitioning between these phases shifts with temperature and pressure. Hong et al. [12] reported that cyclopentane dissolves in substantial quantities within the polyurethane polymer matrix and redistributes between the gas and polymer phases in response to temperature changes. Schumacher et al. [6] experimentally confirmed that cell gas composition in PUR foam changes gradually over hundreds of days following manufacture, governed by the blowing agent solubility and diffusion coefficient within the polymer. The gas–polymer inter-phase redistribution mechanism described in these studies may operate in the present specimens, where n-pentane serves as the blowing agent. Furthermore, the mass–thermal conductivity mismatch observed under standard conditions is directionally consistent with the possibility that such redistribution may be occurring. Neither of these studies, however, reported a transient decrease in thermal conductivity following cutting. The TTCM is therefore a behavior not previously documented for closed-cell foam insulation, and the material conditions that give rise to it are examined in the following analysis.

A physical condition specific to the present specimens makes this mechanism particularly plausible. The boiling point of n-pentane is 36.07 °C, within 3 °C of the HFM hot plate temperature of 33 °C [17]. According to Hong et al. [12], a rise in temperature shifts the equilibrium of blowing agent in a polymer–blowing agent system toward desorption from the polymer phase. Near the boiling point, vapor pressure rises steeply, and this equilibrium shift is expected to be especially pronounced; the tendency for n-pentane remaining in the polymer matrix to transfer into the gas phase would accordingly be enhanced.

The reason this redistribution can lead to a decrease in thermal conductivity lies in the difference in thermal conductivity between n-pentane gas and air. The thermal conductivity of gaseous n-pentane is approximately 0.013–0.015 W/(m·K) [31], lower than that of air, approximately 0.026 W/(m·K). When n-pentane redistributes from the polymer phase into the closed-cell gas phase, the average thermal conductivity of the gas mixture within the cells may decrease accordingly. This process can contribute to a reduction in thermal conductivity even without any mass loss to the exterior, and its direction is consistent with the weight–thermal conductivity discrepancy observed under standard conditions. Prior studies, however, while reporting interphase redistribution and changes in cell gas composition in polyurethane foam, have not reported a transient decrease in thermal conductivity immediately following cutting. In this respect, TTCM represents a novel observation not previously reported in the closed-cell foam literature. It should be noted, however, that this redistribution mechanism has not been directly verified through in situ measurement of cell gas composition, and the present interpretation instead rests on two indirect lines of evidence: the mass–thermal conductivity discrepancy observed under standard conditions, and the proximity of the n-pentane boiling point to the hot-plate temperature.

Residual moisture may also contribute as a secondary factor. Resole resins cure through an acid-catalyzed reaction between phenol and formaldehyde, and water generated as a by-product of this reaction is known to remain within the cells [29]. The thermal conductivity of water vapor is approximately 0.016 W/(m·K) [32], higher than that of n-pentane gas (approximately 0.013–0.015 W/(m·K)) but lower than that of air (approximately 0.026 W/(m·K)). If residual moisture vaporizes under the HFM measurement condition (mean temperature 23 °C), its contribution would act in the direction opposite to the thermal conductivity decrease caused by n-pentane redistribution. Whether moisture contributes additionally to the decrease in thermal conductivity or instead acts to partially offset the redistribution effect cannot be determined from the present data alone. Quantitatively separating these two contributions is left as a subject for future work.

The preceding discussion has examined the driving mechanism of TTCM under standard conditions. Under high-temperature accelerated conditions (H70, H110), the TTCM interval was accompanied by substantial mass loss, reaching approximately 7% in the H70 specimen and 11–14% in the H110 specimen. This contrasts with the −0.2% observed under standard conditions. To identify the cause of this mass loss, we first examined the possibility of thermal decomposition of the resin.

**Figure 5:**
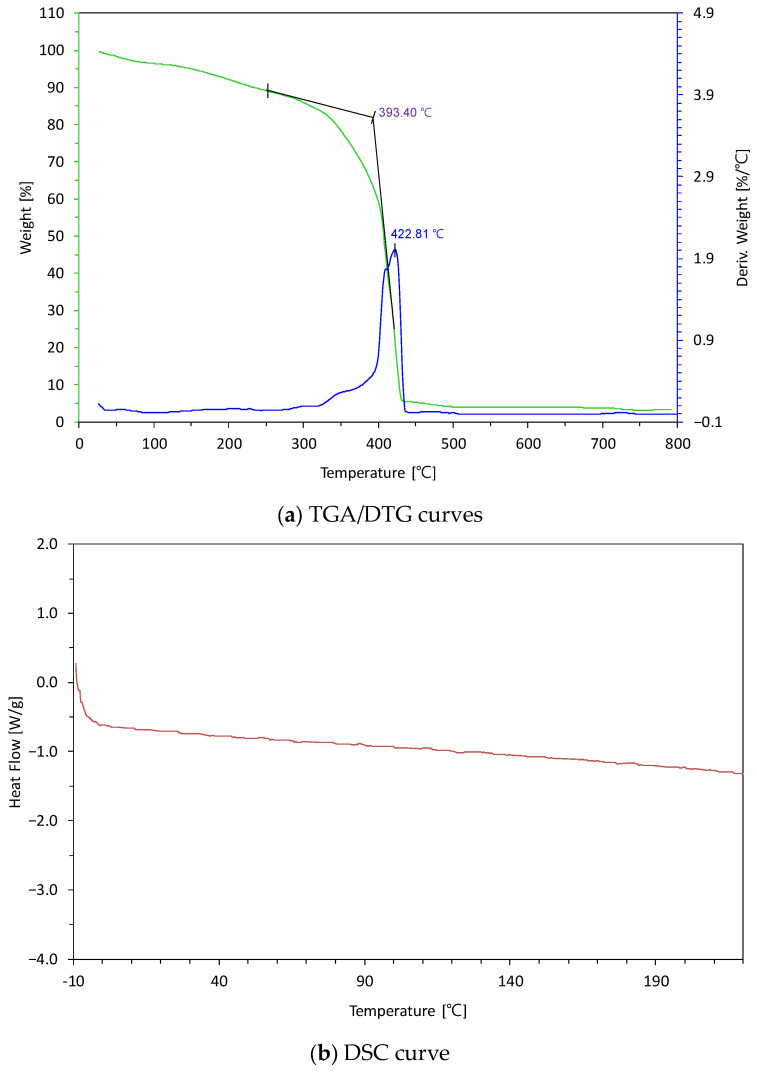
Thermal analysis of Lot-C specimen. (**a**) TGA/DTG curves measured under air atmosphere (30–800 °C, 20 °C/min). (**b**) DSC curve (−10–230 °C, 10 °C/min, second heating).

Figure 5a shows the TGA and DTG curves for the Lot-C specimen. The decomposition onset temperature (Tonset), defined by the intersection of two tangent lines on the DTG curve, is 393.40 °C, with the DTG peak at 422.81 °C. Chen et al. [18] reported that thermal decomposition of PF resins initiates with cleavage of methylene bridges, a process that proceeds above 350 °C. Costa et al. [19] similarly confirmed that the thermal stability of the PF resin skeleton is maintained by the crosslinked methylene bridge structure.

The DSC curve in Figure 5b showed no inflection in heat flow attributable to a glass transition across the entire measurement range of −10 °C to 230 °C, indicating that the glass transition temperature (Tg) exceeds the 230 °C measurement limit. Because the accelerated aging specimens (Lot-A-H110, Lot-B-H70) originate from the same manufacturer and product as Lot-C, these results apply to those specimens as well. At 110 °C, the aging temperature is more than 280 °C below Tonset and below Tg, excluding the possibility of resin decomposition or softening during accelerated aging.

With resin thermal decomposition ruled out, the mass loss under elevated-temperature conditions is interpreted as the combined result of residual moisture evaporation and outward release of n-pentane. Residual moisture, generated as a by-product of the acid-catalyzed resole curing reaction, remains within the cells [29] and evaporates more rapidly at elevated temperatures. n-Pentane, which is retained within cells through intracellular phase redistribution under standard conditions, is subject to reduced solubility in the polymer and an increased diffusion coefficient at elevated temperatures, both of which promote outward release [3,12,13]. However, quantitatively separating these two contributions is not possible with the present data alone.

By contrast, the markedly larger TTCM reduction observed under the H110 condition (−15.1%, −16.7%), compared with the standard condition (−9.7%) and the H70 condition (−6.2%, −8.1%), warrants attention. The relatively modest difference between the H70 and standard conditions suggests that temperature dependence alone is insufficient to account for this disparity. Under the H110 condition, both moisture evaporation and n-pentane release proceed more actively than under H70, and the solubility of n-pentane in the polymer decreases further, potentially increasing the amount available for redistribution into the gas phase [12]. Isolating and quantifying the contribution of each factor individually, however, would require in situ monitoring of cell gas composition, which is left as a subject for future work.

### 3.2. Blowing Agent Release and Long-Term Thermal Conductivity Rise

Following the TTCM minimum, thermal conductivity rises continuously over the long term. Section 3.1 established that the TTCM reflects a re-equilibration of cell gas composition, so a different process must be responsible for the behavior observed after the minimum. Section 3.2.1 tracks mass and thermal conductivity simultaneously in the Post-TTCM phase to assess whether the long-term rise is driven by the gradual outward release of n-pentane. As reported by Glicksman [3] and Pilon et al. [17], the timescale for gas diffusion in closed-cell foams scales with the square of the diffusion path length. Building on this, Section 3.2.2 compares specimens from the same production batch under different diffusion path conditions to determine whether the rate of Post-TTCM thermal conductivity rise shifts in the predicted direction.

#### 3.2.1. Weight and Thermal Conductivity Analysis in the Post-TTCM Phase

**Figure 6:**
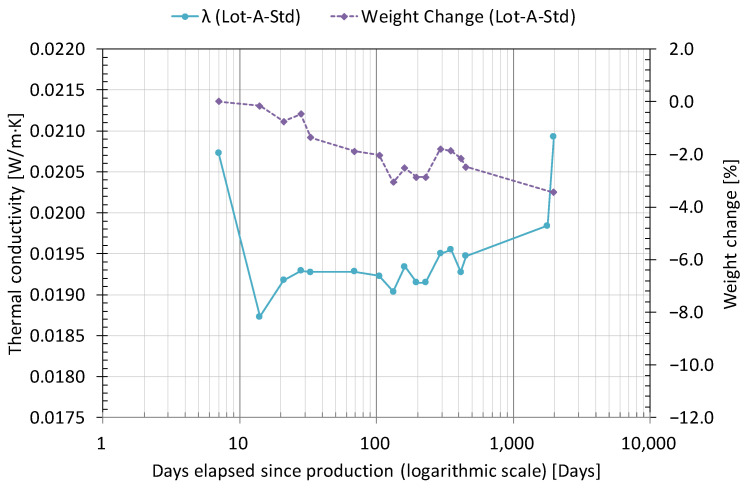
Dual-axis time series of thermal conductivity (λ) and mass change for Lot-A-Std under standard aging conditions, plotted on a logarithmic time scale.

To identify the process driving the thermal conductivity rise in the Post-TTCM phase, the long-term behavior of the standard-condition specimen (Lot-A-Std) is examined first. Starting from 0.0207 W/(m·K) on Day 7, Lot-A-Std reached its TTCM minimum of 0.0187 W/(m·K) on Day 14 and then increased gradually to 0.0209 W/(m·K) over the following 1964 days. During the same period, mass decreased by 3.30%. That thermal conductivity continued to rise steadily over this extended period, despite only a minor change in weight, suggests that the cell gas composition continues to change slowly even under the standard 23 °C condition. Figure 6 presents this behavior on a dual axis.

This behavior is consistent with results reported in the same direction for PUR and PIR foams. Andersons et al. [10] quantified the effective diffusion coefficients of blowing agents, including n-pentane, by back-calculating them from thermal conductivity aging data, while Berardi and Madzarevic [11] used GC-FID analysis to directly confirm that a decrease in n-pentane concentration is associated with an increase in thermal conductivity in PIR foam. These prior studies, however, were conducted on PUR and PIR foams, and whether the same mechanism operates in PF foam requires separate verification. To examine this, we investigated the relationship between weight and thermal conductivity in greater detail under conditions where blowing agent release is accelerated.

**Figure 7:**
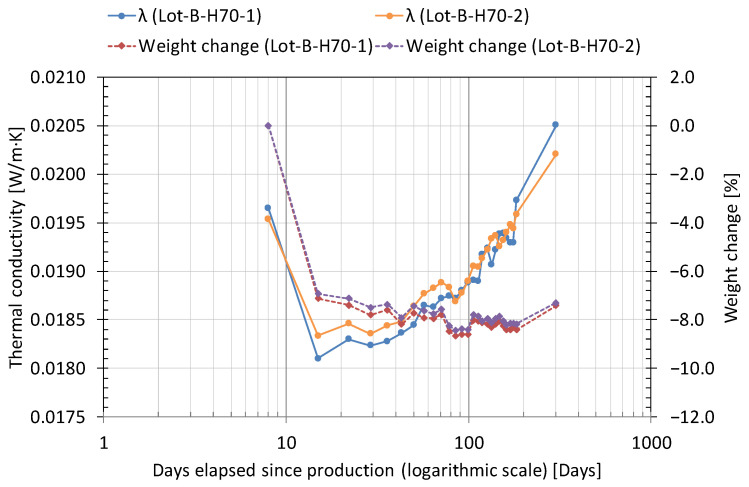
Dual-axis time series of thermal conductivity (λ) and mass change for Lot-B-H70-1 and Lot-B-H70-2 under 70 °C accelerated aging, plotted on a logarithmic time scale.

Under the 70 °C accelerated condition, a pattern distinct from that of the standard condition emerged. Table 5 summarizes the TTCM minimum conditions for all specimens, and Table 6 compares key values for the TTCM and Post-TTCM phases of Lot-B-H70-1 and Lot-B-H70-2. Figure 7 presents the mass change and thermal conductivity of both specimens on a dual axis.

Table 6 quantitatively demonstrates that the TTCM and Post-TTCM intervals proceed in markedly different manners in both specimens. Over the seven-day TTCM interval, both specimens lost approximately 7% of their weight. During the subsequent 287-day Post-TTCM interval, by contrast, weight change amounted to only 0.3–0.4%, while thermal conductivity rose an additional 13.3% in Lot-B-H70-1 and 10.4% in Lot-B-H70-2. The fact that thermal conductivity continued to rise while weight remained essentially unchanged is consistent with the interpretation that the two intervals are governed by distinct driving processes.

In Lot-B-H70-1, this transition became particularly evident around Day 85. Between Day 15 and Day 85, weight decreased from 193.2 g to 189.9 g while thermal conductivity rose concurrently. After Day 85, however, weight stabilized within the range of 189.9 to 191.3 g, a level consistent with measurement error, whereas thermal conductivity continued to rise from 0.0181 to 0.0197 W/(m·K). This trend is consistent in direction with the behavior observed in Lot-B-H70-2.

This behavior is explained by the gradual replacement of n-pentane within the cells by air. The mass released as n-pentane is displaced is exceedingly small relative to the total mass of the foam and therefore falls below the resolution of weight measurement. The gradual replacement of highly insulating n-pentane (approximately 0.013–0.015 W/(m·K)) by air (approximately 0.026 W/(m·K)), however, is directly reflected in an increase in thermal conductivity [31]. In other words, the continued rise in thermal conductivity despite stagnant weight points to ongoing replacement of n-pentane by air during the Post-TTCM interval. Even where the magnitude of this change is too small to be captured by weight measurement, the resulting change in cell gas composition is sensitively reflected in thermal conductivity.

Between Day 183 and Day 302, a weight increase of 2.1 g and 1.9 g, respectively, was observed in both specimens. Thermal conductivity, however, continued to rise concurrently over this interval. The possibility that this weight increase results from moisture reabsorption from the measurement environment cannot be excluded. Because the thermal conductivity of water vapor (approximately 0.016 W/(m·K)) is lower than that of air (approximately 0.026 W/(m·K)) [32], however, moisture absorption should, if anything, act to lower thermal conductivity. The fact that thermal conductivity nonetheless rose during this interval suggests that the replacement of n-pentane by air continued throughout this period as well, and that its effect outweighed the reduction in thermal conductivity associated with moisture absorption. Quantitatively separating the contributions of these two factors is not possible with the present data alone, and is left as a subject for future work.

Interpretation is somewhat more complex under the high-temperature accelerated condition (H110). As shown in Table 5, the H110 specimens had already undergone a weight loss ranging from 11.7 to 14.2% during the TTCM interval. Although the possibility of thermal decomposition of the resin was excluded in Section 3.1.2, the respective contributions of moisture evaporation and n-pentane release to this weight loss cannot be separated from the present data. Furthermore, the trend in weight change during the Post-TTCM interval following the TTCM minimum is difficult to assess, as the observation period was limited to only 33 days. The direction of the increase in thermal conductivity, however, is consistent with that observed in Lot-B-H70 and Lot-A-Std, suggesting that the same mechanism is operating, albeit at a rate that varies with temperature.

#### 3.2.2. Release Path Dependence

The results of Section 3.2.1 support the interpretation that gradual outward release of n-pentane drives the thermal conductivity rise in the Post-TTCM phase. According to Fick’s law of gas diffusion, changes in diffusion path conditions are expected to be reflected in the rate of release and in the associated thermal conductivity behavior. This section compares two specimens from the same production batch (Lot-C) prepared under differing diffusion path conditions and examines whether the measured data are directionally consistent with this expectation.

n-Pentane exists in a dissolved state within the polymer matrix, then desorbs and migrates into the cell gas phase, and subsequently permeates through the cell walls to be released outward [12]. According to Pilon et al. [17], this release rate depends strongly on the length of the permeation path; in closed-cell foams, the time required for cell gas to reach the exterior scales with the square of the path length.

GC-FID results from the CP series, specimens cut from the same lot (Lot 240529) after different sealed storage durations, provide indirect directional support for this. CP-0, CP-34, and CP-105 were cut from the intact board (80 mm) at 0, 34, and 105 days after production, respectively, double-sealed, and analyzed by GC-FID 469–568 days after cutting. In all three specimens, n-pentane had reached levels below the GC-FID detection limit. By contrast, CP-544, cut 544 days after production and analyzed four days after cutting, still contained 99–124 mg/L of n-pentane. The difference between the two groups lies in the time elapsed since cutting, confirming that release progresses with time. The CP series, however, varies only in storage duration, not the path condition itself, within a fixed board thickness (80 mm). The influence of path length is examined directly through the thermal conductivity behavior of Lot-C-Slicing and Lot-C-Std.

**Figure 8:**
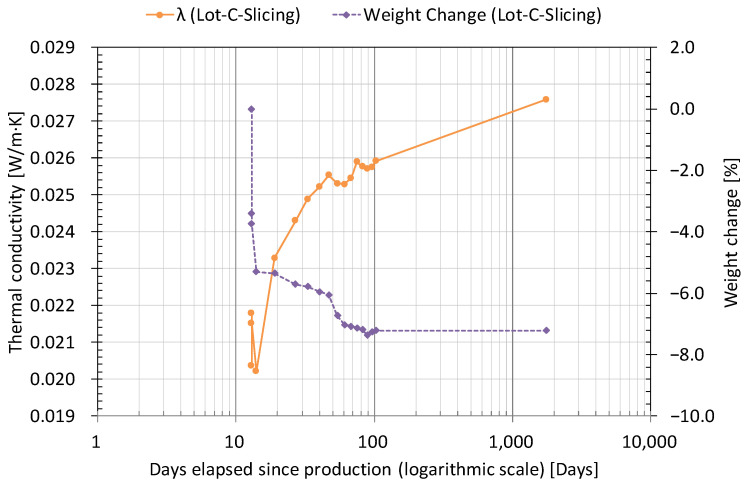
Dual-axis time series of thermal conductivity (λ) and mass change for Lot-C-Slicing under slicing-accelerated aging (KS M ISO 11561 Method B), plotted on a logarithmic time scale.

As summarized in Table 7, Lot-C-Slicing was prepared by slicing the original board to approximately 10 mm with facings removed, substantially shortening the diffusion path. After its TTCM minimum of 0.0202 W/(m·K) on Day 14, thermal conductivity rose to 0.0259 W/(m·K) within 89 days, an increase of 28.2%. Mass continued to decrease from its value immediately after cutting during this interval. The concurrent mass reduction and thermal conductivity rise parallel the behavior of H70 specimens described in Section 3.2.1. Between Day 54 and Day 103, thermal conductivity stabilized in the range of 0.0253–0.0259 W/(m·K), and mass change also plateaued at the same time. Figure 8 presents this behavior on a dual axis, visually distinguishing the accelerated release phase from the stabilization phase.

Lot-C-Std, from the same production batch as Lot-C-Slicing, was aged under standard conditions with its aluminum foil facing intact. Thermal conductivity showed no meaningful change from its TTCM minimum of 0.0204 W/(m·K) on Day 13 to 0.0200 W/(m·K) on Day 1811, a span of 1798 days. CP-544 GC-FID results similarly detected n-pentane at 113–124 mg/L in the upper zone, 99–104 mg/L in the central zone, and 114–123 mg/L in the lower zone, demonstrating that n-pentane can persist over the long term under conditions with intact facings. However, these results reflect the combined effect of board thickness, bilateral facing sealing, and the short time elapsed since cutting, and cannot be attributed to facing effect alone.

The contrasting long-term thermal conductivity evolution of Lot-C-Slicing and Lot-C-Std supports the interpretation established in Section 3.2.1. Under the shortened-path condition, thermal conductivity rose by 28.2% within 89 days; under the blocked-path condition, it was effectively unchanged over 1798 days. Together, these results support the conclusion that the thermal conductivity rise in the Post-TTCM phase is sensitive to diffusion path conditions. Quantitative determination of the effective diffusion coefficient would require measurement of cell size, cell wall thickness, and closed-cell fraction at each position, which lies beyond the scope of the present data and is left for future investigation.

### 3.3. Thickness-Direction Position Dependence

In Section 3.2.2, an additional rise in thermal conductivity was observed in Lot-C-Slicing following the stabilization of release, but the governing factors cannot be identified from the present data alone. This section examines, in an exploratory manner, blowing agent distribution, chemical structure, and thermal conductivity behavior as a function of through-thickness position. Section 3.2 established that diffusion path length governs the rate of blowing agent release. Within the board, the distance to the outer surface varies by through-thickness position; consequently, blowing agent distribution, release rate, and the extent of inward air infiltration may all differ by position. Section 3.3.1 presents through-thickness distributions of residual blowing agent and resin chemical aging indicators, while Section 3.3.2 addresses position-dependent thermal conductivity behavior. The specimens examined in the two subsections differ, but all originate from the same manufacturer and product. The sections collectively assess whether the trends observed from each perspective align in a consistent direction along the shared axis of through-thickness position.

#### 3.3.1. Through-Thickness Blowing Agent Distribution and Chemical Aging Indicators

To examine how thickness-direction position is reflected in the physical and chemical state of the foam, this section reviews two independent lines of analysis: the thickness-direction distribution of the blowing agent as determined by GC-FID, and the thickness-direction chemical aging indicators obtained via ATR-FT-IR. Although blowing agent distribution and chemical aging are indicators of a fundamentally different nature, if both exhibit a consistent trend along the thickness direction, this lends support to thickness-direction position being a variable that exerts a genuine influence.

The GC-FID analysis of specimen CP-544, a sealed full-thickness board specimen, revealed residual n-pentane concentrations of 113–124 mg/L in the upper region, 99–104 mg/L in the middle region, and 114–123 mg/L in the lower region. The higher concentrations in the upper and lower zones relative to the central zone indicate that the residual blowing agent distribution is not uniform across the board thickness, even within a single board. Whether this distribution reflects an initial concentration gradient established during foaming and curing, or one that developed through gas redistribution during long-term storage, cannot be resolved from a single time-point measurement. Because regional cell structure was not directly measured, this interpretation remains qualitative.

To examine through-thickness changes in chemical structure following long-term aging, ATR-FT-IR analysis was performed on three specimens. A-Core is a sample from the core region of Lot-A with 1932 days of aging; B-Surf and C-Surf are surface-region samples from Lot-B and Lot-C with 1889 and 1697 days of aging, respectively. The sealed CP-544 specimen was also analyzed as an unaged reference. Peak intensities were normalized against the aromatic ring C=C stretching vibration at 1600 cm^−1^, a band that remains relatively stable through curing and long-term aging [29,30], and the results are summarized in Table 8.

I_815_ corresponds to the C-H out-of-plane bending vibration of para-disubstituted benzene rings in the cured resole resin and reflects the degree to which the para-crosslinked structure is preserved through methylene bridges [19,29]. I_815_ for the unaged reference CP-544 is 0.917; A-Core shows a modest decrease to 0.879, a decline of only 4.1%. The surface-region specimens B-Surf and C-Surf, however, show values of 0.407 and 0.559, respectively, substantially lower than the core, indicating a markedly lower degree of crosslink structure retention at the surface.

I_1650_ corresponds to the stretching vibration of carbonyl groups formed through oxidation of methylene bridges and serves as an indicator of oxidative degradation of the resin [18]. The core specimen A-Core, at 1.085, exceeds the unaged reference value of 0.936 only slightly, whereas the surface specimens show higher values, 1.327 for B-Surf and 1.087 for C-Surf. I_1475_, corresponding to the CH2 scissoring vibration of methylene bridges, serves as a complementary indicator of crosslinked structure [29,30] and is lower at the surface than at the core. All three indices thus change in the same direction at the surface, namely, a reduction in crosslinked structure accompanied by progressing oxidation.

Although the resin type differs, the same thickness-direction pattern, in which the crosslinked structure of the core is better preserved than that of the surface, has also been reported in PIR foam. According to Reignier et al. [26], the isocyanurate crosslink content at the surface of PIR foam was, on average, 2.69 times lower than that at the core, a difference attributed to the core-surface temperature gradient arising from heat loss at the surface during manufacturing. The reduced crosslink preservation and progressed oxidation observed in the surface specimens of the present study align with this core-surface crosslinking gradient pattern.

All three specimens were taken from the same product manufactured by the same manufacturer. Although the possibility of batch-to-batch variation cannot be entirely excluded, the fact that the two independently sampled surface specimens, B-Surf and C-Surf, consistently show the same direction of change across all three indices, namely, a decrease in I_815_, an increase in I_1650_, and a decrease in I_1475_, indicates that the observed trend is difficult to attribute to individual specimen variation alone. However, because this analysis is based on a single-time-point measurement, the observed differences in chemical structure cannot be definitively attributed to thickness-direction position, batch variation, or other factors.

The two observations presented in this section, namely, the blowing agent distribution obtained through GC-FID and the chemical aging indicators obtained through ATR-FT-IR, exhibited a consistent directional trend with respect to thickness-direction position. Whether this directional trend also extends to thermal conductivity behavior is discussed jointly in Section 3.3.2.

#### 3.3.2. Position-Dependent Thermal Conductivity Evolution

**Figure 9:**
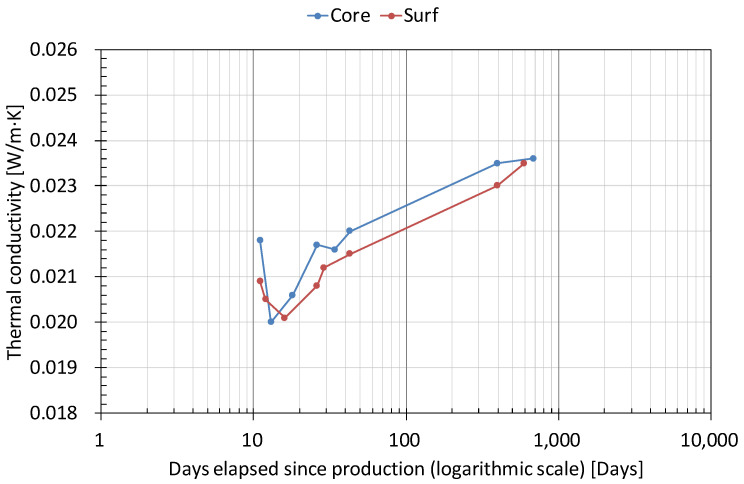
Thermal conductivity evolution of core-region (Core) and surface-region (Surf) specimens from the same production lot, plotted on a logarithmic time scale.

To examine whether through-thickness position is reflected in thermal conductivity behavior, Core specimens, taken from the central region of Lot 240529, were compared with Surf specimens taken from the surface region beneath the aluminum foil facing. The two specimen types share the same cutting date, facing removal date, thickness, and measurement schedule, allowing positional effects to be compared without confounding batch differences. Figure 9 presents the thermal conductivity time series of the two specimens on a logarithmic time scale.

As shown in Figure 9, the initial thermal conductivity differed between the two specimens. On Day 11, Core measured 0.0218 W/(m·K) and Surf measured 0.0209 W/(m·K). Both specimens subsequently reached virtually the same TTCM minimum: Core at 0.0200 W/(m·K) on Day 13, and Surf at 0.0201 W/(m·K) on Day 16. The difference in TTCM reduction magnitude between the two specimens thus reflects their differing initial values rather than any difference in the minimum reached. The origin of the initial value difference cannot be determined from the present data alone; however, this positional difference in initial state is directionally consistent with the non-uniform through-thickness blowing agent distribution observed in Section 3.3.1.

Over the long term, the thermal conductivity of both specimens converges to similar levels: Core reaches 0.0236 W/(m·K) on Day 684, and Surf 0.0235 W/(m·K) on Day 595. The direction of thermal conductivity rise after the TTCM minimum is the same in both specimens, consistent with the progressive blowing agent release behavior described in Section 3.2.1. The positional difference in initial state appears to diminish over time.

The two observations in this section collectively show, at a directional level, that blowing agent distribution, resin chemical structure, and initial thermal conductivity may all vary together as a function of through-thickness position. However, because the data from the three perspectives were obtained from different specimens and the chemical analysis was limited to a single time point, it is not possible to connect positional physical state, chemical aging, and thermal conductivity behavior into a single causal chain. Bridging this gap would require future studies in which core and surface samples are taken from the same batch simultaneously and tracked together in both chemical structure and thermal conductivity over time, with blowing agent distribution, oxidation indicators, and thermal conductivity measured in an integrated manner from the same specimen at each through-thickness position.

## 4. Limitations

This study has established the existence of TTCM and its dependence on closed-cell structural integrity through four independent lines of evidence. The elucidation of the mechanism driving this phenomenon and the exploration of its thickness-direction position dependence, however, represent only a first exploratory step, and the limitations set out below stem from this exploratory scope.

The redistribution of n-pentane within the cells, proposed as the driving mechanism of TTCM, constitutes an exploratory interpretation grounded in two circumstantial lines of evidence: the mass–thermal conductivity discrepancy observed under standard conditions and the proximity of the n-pentane boiling point to the hot-plate temperature. Direct verification through in situ measurement of cell gas composition, together with the quantitative separation of the contribution of residual moisture from that of the redistribution effect, is left to subsequent confirmatory studies. The same holds for disentangling the respective contributions of moisture evaporation and n-pentane release to the mass loss observed under high-temperature accelerated conditions.

This exploratory character is also reflected in the experimental design. For most conditions, thermal conductivity and weight were tracked using a single specimen, reflecting a design choice that prioritized confirming the existence and directionality of TTCM across a broad range of conditions over obtaining statistically replicated data within a narrower set of conditions. For the H70 and H110 conditions, however, two nominally replicate specimens were available (Table 5); the TTCM reduction magnitude differed between these replicates by 1.6–1.9 percentage points, a spread considerably smaller than the differences observed between conditions (e.g., standard versus H110). While this limited comparison cannot substitute for full statistical replication, it lends some support to the interpretation that the larger differences reported between conditions reflect the aging condition itself rather than specimen-level variability alone. Statistical verification through replicate specimens across all conditions constitutes the next step, one that can be pursued within a narrower range of conditions informed by the present exploratory findings.

The discussion of thickness-direction position dependence carries the same character. The three observations, namely blowing agent distribution (GC-FID), chemical aging indicators (ATR-FT-IR), and thermal conductivity behavior, were obtained from different specimens and at different time points, and the fact that they exhibit a consistent directional trend lends exploratory support to the possibility that thickness-direction position influences multiple properties.

Subsequent confirmatory research could proceed along two directions. One would involve monitoring cell gas composition in situ to directly verify the redistribution mechanism and to separate the contribution of moisture. The other would involve jointly sampling core and surface specimens from the same production batch and tracking blowing agent distribution, chemical structure, and thermal conductivity in an integrated manner, thereby establishing the causal relationship underlying thickness-direction position dependence.

## 5. Conclusions

This study consistently observed, across numerous specimens and aging conditions, that the thermal conductivity of phenolic foam (PF) insulation declines over a period of days to weeks immediately after cutting, passes through a minimum, and subsequently rises again. We define this initial decline–minimum–rise pattern as the transient thermal conductivity minimum (TTCM). This finding indicates that the conventional assumption of a monotonic increase in thermal conductivity following cutting does not hold, at least during the early period, for the specimens examined in this study. The premise of this study is that an outwardly simple time-dependent change in thermal conductivity conceals a process that requires separate characterization.

Under identical HFM measurement conditions, the magnitude of the TTCM reduction varied by more than fourfold across specimens, ranging from 3.8% to 16.7%. TTCM occurred in the Board specimen, in which the aluminum foil facing remained intact, whereas it was never observed under any condition in the reference PF foam with cell-wall micro-perforations (Ref-OC). Together, these two results are consistent with the interpretation that an intact closed-cell structure is a necessary condition for TTCM to occur. This condition alone, however, does not explain why TTCM appears within this particular temperature range; it is interpreted as arising in conjunction with a physical property of the blowing agent, n-pentane, whose boiling point lies close to the test temperature. Taken together, these observations indicate that the occurrence and magnitude of TTCM emerge only when the physical condition of an intact closed-cell structure combines with the chemical condition of the blowing agent’s phase-change behavior. This perspective may also serve as a basis for examining whether analogous behavior in closed-cell foams blown with different agents would appear within a different temperature range, or fail to appear at all.

Under standard conditions, mass changed by only −0.2%, whereas thermal conductivity decreased by 9.7%, a difference in magnitude of roughly fiftyfold between the two quantities. This suggests that even an internal change too small to be detected gravimetrically may be reflected in thermal conductivity. When assessing the early-stage aging of closed-cell foam insulation, thermal conductivity measurement can therefore serve as a complementary indicator, capturing internal changes that mass tracking alone would likely miss.

Under the 70 °C accelerated condition, mass change remained negligible over the 287-day Post-TTCM period, yet thermal conductivity rose by more than 10%. The same trend was confirmed in a comparison of specimens from the same production batch under different diffusion-path conditions. The sliced specimen, with a shortened diffusion path, showed a 28.2% rise within 89 days, whereas the standard-condition specimen with the facing intact showed no meaningful change over 1798 days. In this comparison, the rate of thermal conductivity rise during the Post-TTCM phase differed by more than an order of magnitude between the two diffusion-path conditions examined. This points to the long-term rise in thermal conductivity following the TTCM minimum as originating from a process distinct from TTCM itself. Accordingly, when comparing accelerated aging results obtained under different diffusion-path conditions with one another, or when extrapolating them to actual field conditions, differences in path condition must be taken into account.

An exploratory analysis of through-thickness position dependence found that three independent lines of evidence, namely residual blowing agent concentration, resin chemical structure, and initial thermal conductivity, all pointed in the same direction, suggesting that the extent of aging can differ by sampling location even within a single board.

The TTCM phenomenon identified in this study, together with the interpretation that TTCM and Post-TTCM arise from distinct processes, contributes to the understanding of the early-stage and long-term thermal behavior of PF insulation. It provides an experimental basis for future research on the long-term thermal performance of closed-cell polymeric foam insulation.

## Figures and Tables

**Table 1:** Long-term and accelerated aging specimens (Lots A, B, and C).

Specimen	Production Date (Lot)	Aging Condition	Facing Status	Thickness (mm)	Mass Tracking	Analysis
Lot-A-Std	2020-08-27 (A)	Standard (23 °C)	Removed	66	Yes	λ, Mass
Lot-A-H110-1		110 °C Accelerated	Removed	66	Yes	λ, Mass
Lot-A-H110-2		110 °C Accelerated	Removed	66	Yes	λ, Mass
A-Core		Standard—core region	Removed	—	—	ATR-FT-IR
Lot-B-H70-1	2020-10-14 (B)	70 °C Accelerated	Removed	65	Yes	λ, Mass
Lot-B-H70-2		70 °C Accelerated	Removed	65	Yes	λ, Mass
B-Surf		Standard—surface region	Removed	—	—	ATR-FT-IR
Lot-C-Std	2021-04-24 (C)	Standard (23 °C)	Retained	62	Yes	λ, Mass, TGA, DSC
Lot-C-Slicing		Slicing (KS M ISO 11561)	Removed	~66 (sliced; restacked from 72 mm)	Yes	λ, Mass
C-Surf		Standard—surface region	Removed	—	—	ATR-FT-IR

**Table 2:** Lot 240529 specimens (produced 29 May 2024) for thermal conductivity tracking and blowing agent analysis.

Specimen	Sampling Location	Facing Status	Sealed Storage Prior to Cutting (Days)	Analysis
Core	Central region	Removed	0	λ, Mass
Surf	Foil-faced surface region	Removed	0	λ, Mass
Board	Full board	Retained	544	λ, Mass
CP-0	Full board, thickness- direction sectioning	Retained	0	GC-FID
CP-34	Full board, thickness- direction sectioning	Retained	34	GC-FID
CP-105	Full board, thickness- direction sectioning	Retained	105	GC-FID
CP-544	Full board, thickness- direction sectioning	Retained	544	GC-FID

**Table 3:** Reference PF specimens (produced 28 April 2021).

Specimen	Aging Condition	Facing Status	Thickness (mm)	Mass Tracking	Analysis
Ref-OC-Std	Standard (23 °C)	Removed	50	Yes	λ, Mass, SEM
Ref-OC-Slicing	Slicing (KS M ISO 11561)	Removed	~56 (sliced; restacked from 61.2 mm)	Yes	λ, Mass

**Table 4:** Quantification of the TTCM reduction across all conditions for thick specimens, measured under identical HFM operating conditions (hot plate 33 °C, cold plate 13 °C, mean temperature 23 °C). (For Board, values in parentheses indicate days elapsed since cutting).

Specimen	Thickness [mm]	Initial Thermal Conductivity [W/(m·K)] (a)	Min. Thermal Conductivity [W/(m·K)] (b)	Reduction ((b)/(a) − 1) [%]	Days to Min. [Day Since Prod.]
Board	80	0.0207 (Day 544)	0.0199 (Day 558)	−3.9	558 (14)
Surf	50	0.0209 (Day 11)	0.0201 (Day 16)	−3.8	16
Core	50	0.0218 (Day 11)	0.0200 (Day 13)	−8.3	13
Lot-A-Std	66	0.0207 (Day 7)	0.0187 (Day 14)	−9.7	14
Lot-B-H70-1	65	0.0197 (Day 8)	0.0181 (Day 15)	−8.1	15
Lot-B-H70-2	65	0.0195 (Day 8)	0.0183 (Day 15)	−6.2	15
Lot-A-H110-1	66	0.0216 (Day 7)	0.0180 (Day 14)	−16.7	14
Lot-A-H110-2	66	0.0212 (Day 7)	0.0180 (Day 14)	−15.1	14

**Table 5:** TTCM minimum conditions for specimens under standard and accelerated aging.

Specimen	Initial λ [W/(m·K)]	Min. λ [W/(m·K)]	λ Change [%]	Initial Weight [g]	Weight at Min. λ [g]	Weight Change [%]	Days to Min. λ
Lot-A-Std	0.0207 (Day 7)	0.0187 (Day 14)	−9.7	214.4	214.1	−0.2	14
Lot-B-H70-1	0.0196 (Day 8)	0.0181 (Day 15)	−7.7	208.0	193.2	−7.1	15
Lot-B-H70-2	0.0195 (Day 8)	0.0183 (Day 15)	−6.2	216.1	201.2	−6.9	15
Lot-A-H110-1	0.0216 (Day 7)	0.0180 (Day 14)	−16.7	217.6	192.2	−11.7	14
Lot-A-H110-2	0.0212 (Day 7)	0.0180 (Day 14)	−15.1	222.9	191.1	−14.2	14

**Table 6:** Phase-by-phase comparison of weight change and thermal conductivity (λ) change for Lot-B-H70-1 and Lot-B-H70-2.

Specimen	Phase	Period	Duration [Days]	Weight Change [%]	λ Change [%]
Lot-B-H70-1	TTCM	Day 8 → Day 15	7	−7.1	−7.7
Lot-B-H70-1	Post-TTCM	Day 15 → Day 302	287	−0.3	+13.3
Lot-B-H70-2	TTCM	Day 8 → Day 15	7	−6.9	−6.2
Lot-B-H70-2	Post-TTCM	Day 15 → Day 302	287	−0.4	+10.4

**Table 7:** Release path conditions and thermal conductivity (λ) evolution in the Post-TTCM phase for Lot-C-Slicing and Lot-C-Std.

Specimen	Release Path Condition	TTCM Min. λ [W/(m·K)]	Post-TTCM λ Change
Lot-C-Slicing	Shortened (~10 mm; facers removed)	0.0202 (Day 14)	+28.2% (Day 14 → 103)
Lot-C-Std	Blocked (Al foil facer intact)	0.0204 (Day 13)	0.0% (Day 13 → 1811)

**Table 8:** ATR-FT-IR normalized peak intensity ratios of PF foam specimens by aging stage and sampling position.

Specimen	Production Date	Age (Days)	Sampling Position	I_815_	I_1650_	I_1475_
CP-544	2024-05-29	544	Core (sealed reference)	0.917	0.936	2.143
A-Core	2020-08-27	1932	Core	0.879	1.085	2.608
B-Surf	2020-10-14	1889	Surface	0.407	1.327	1.708
C-Surf	2021-04-24	1697	Surface	0.559	1.087	2.247

## Data Availability

The original contributions presented in this study are included in the article.
